# Natural Products from the Genus *Tephrosia*

**DOI:** 10.3390/molecules19021432

**Published:** 2014-01-27

**Authors:** Yinning Chen, Tao Yan, Chenghai Gao, Wenhao Cao, Riming Huang

**Affiliations:** 1 Key Laboratory of Plant Resources Conservation and Sustainable Utilization, South China Botanical Garden, Chinese Academy of Sciences, Guangzhou 510650, China; E-Mail: chendianyu3356@163.com; 2 South China Sea Institute of Oceanology, Chinese Academy of Sciences, Guangzhou 510301, China; E-Mails: yantao@scsio.ac.cn (T.Y.); chromo@163.com (W.C.); 3 Guangxi Key Laboratory of Marine Environmental Science, Guangxi Academy of Sciences, Nanning 530007, China; E-Mail: gaochenghai@aliyun.com

**Keywords:** *Tephrosia*, chemical constituents, proposed biosynthetic pathways, synthesis, biological activity

## Abstract

The genus *Tephrosia*, belonging to the Leguminosae family, is a large pantropical genus of more than 350 species, many of which have important traditional uses in agriculture. This review not only outlines the source, chemistry and biological evaluations of natural products from the genus *Tephrosia* worldwide that have appeared in literature from 1910 to December 2013, but also covers work related to proposed biosynthetic pathways and synthesis of some natural products from the genus *Tephrosia*, with 105 citations and 168 new compounds.

## 1. Introduction

The genus *Tephrosia*, belonging to the Leguminosae family, is a large pantropical genus of more than 350 species, many of which have important traditional uses [1,2]. Phytochemical investigations have revealed the presence of glucosides, rotenoids, isoflavones, chalcones, flavanones, flavanols, and prenylated flavonoids [1,2,3,4,5,6,7,8,9] of chemotaxonomic importance in the genus [10]. Moreover, bioactivity has been studied extensively, indicating that chemical constituents and extracts of the genus *Tephrosia* exhibited diverse bioactivities, such as insecticidal [11], antiviral [12], antiprotozoal [13], antiplasmodial [14] and cytotoxic [15] activities. 

So far, the reviews on natural products isolated from the genus *Tephrosia* are limited [16]. To gain a comprehensive and systematic understanding of this genus, this review outlines the chemistry, proposed biosynthetic pathways, synthesis, and biological evaluations of natural products from the genus *Tephrosia* worldwide that have appeared in literature from 1971 to December 2013, with 105 citations and 168 new compounds from them.

## 2. Chemical Constituents

**Figure 1:**
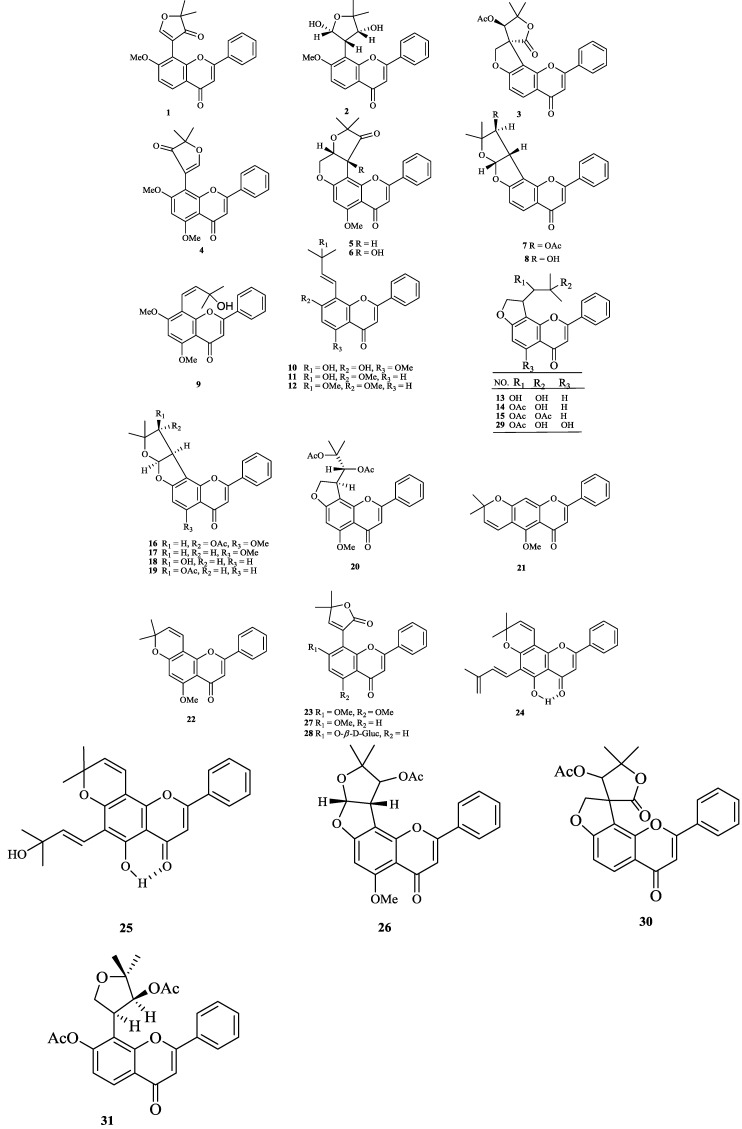
Flavones from genus *Tephrosia*.

**Figure 2:**
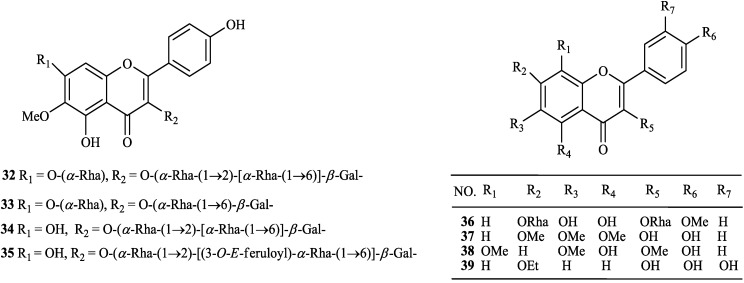
Flavonols from genus *Tephrosia*.

**Figure 3:**
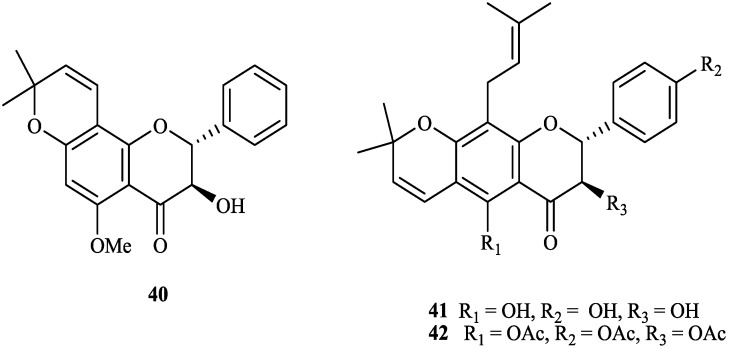
Flavanonols from genus *Tephrosia*.

**Figure 4:**
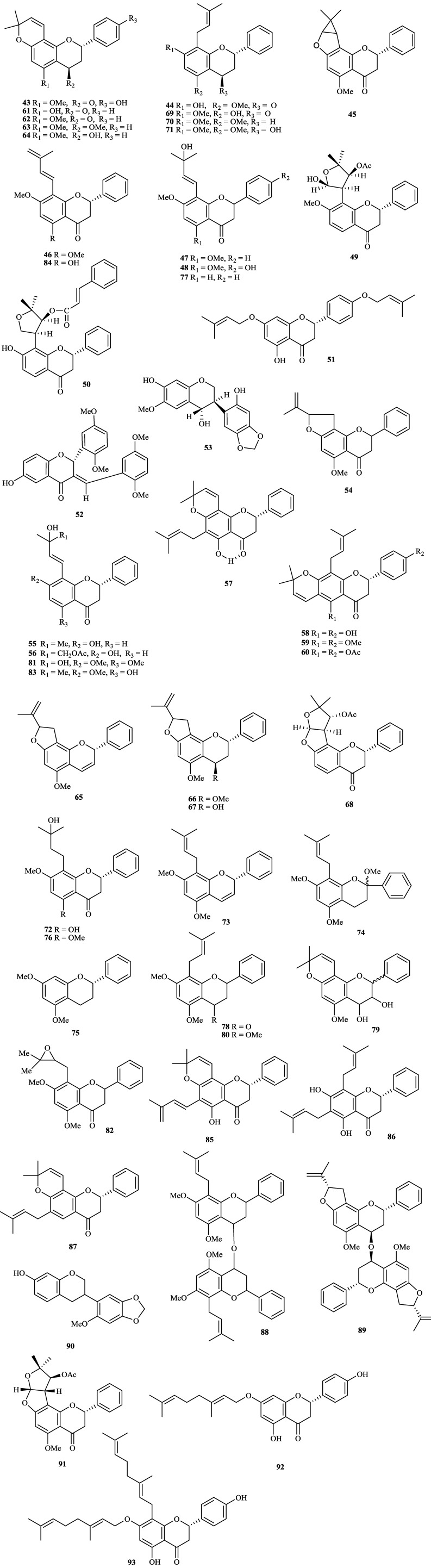
Flavans from genus *Tephrosia*.

**Figure 5:**
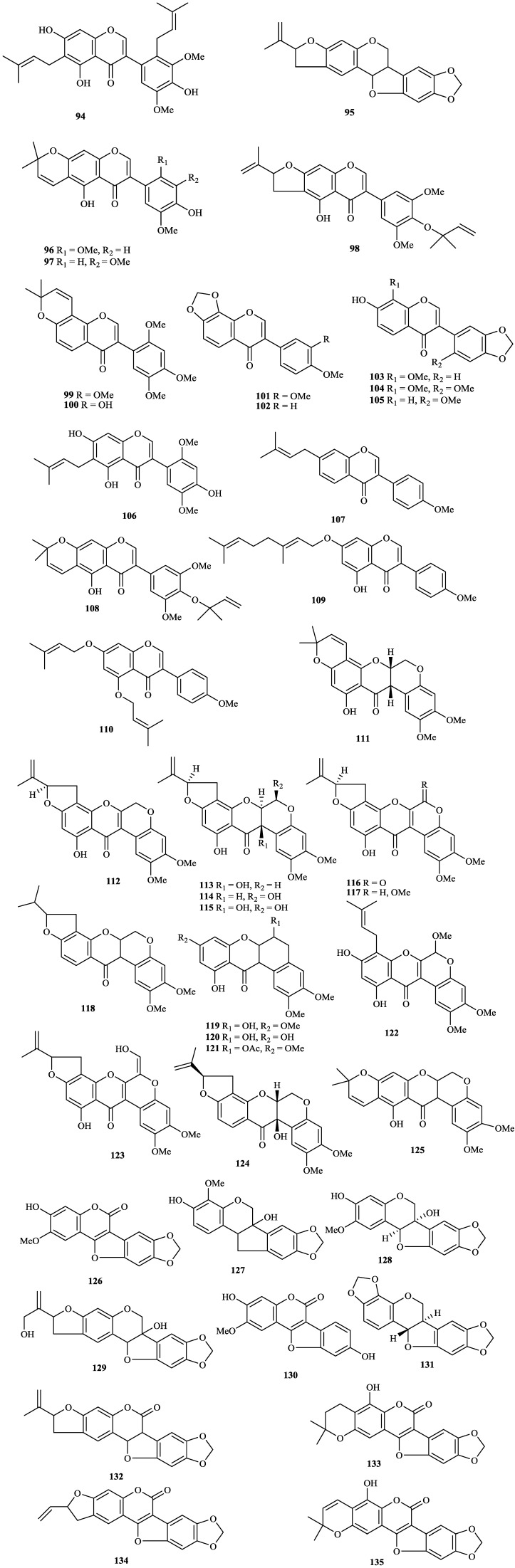
Isoflavones from genus *Tephrosia*.

**Figure 6:**
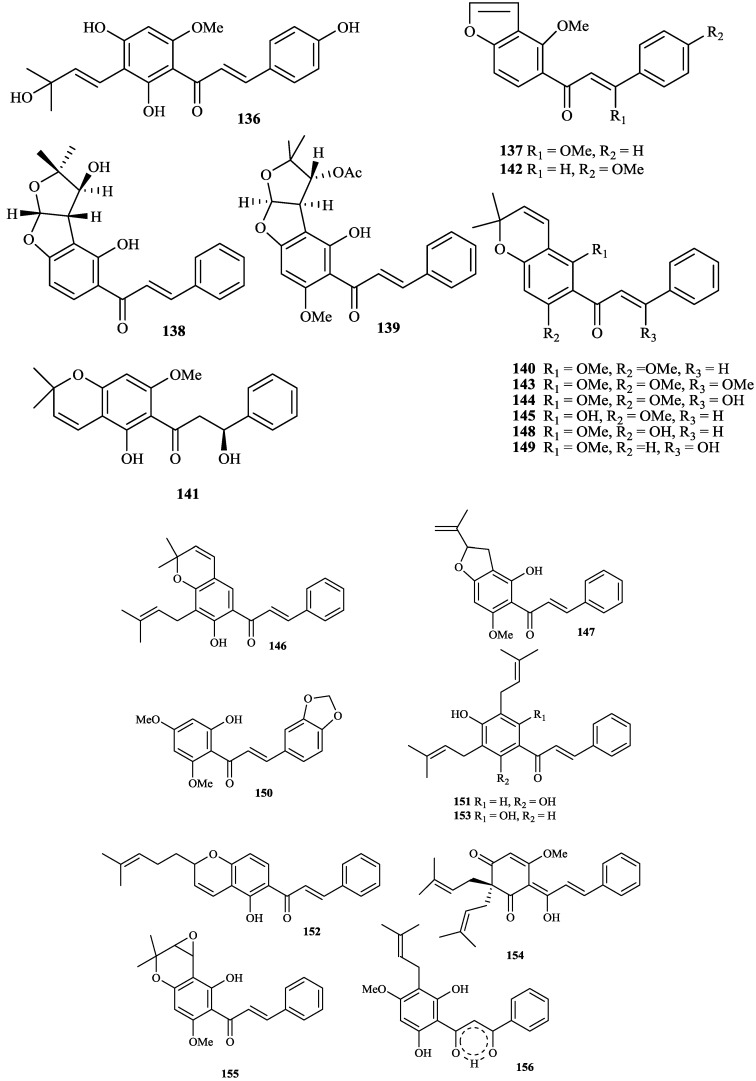
Chalcones from genus *Tephrosia*.

**Figure 7:**
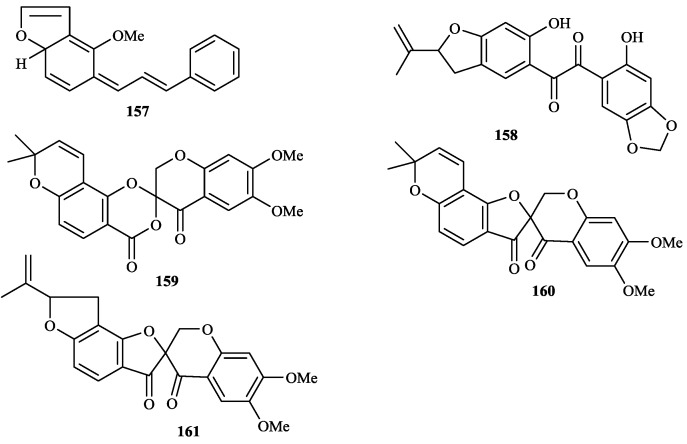
Other flavonoids from genus *Tephrosia*.

**Figure 8:**
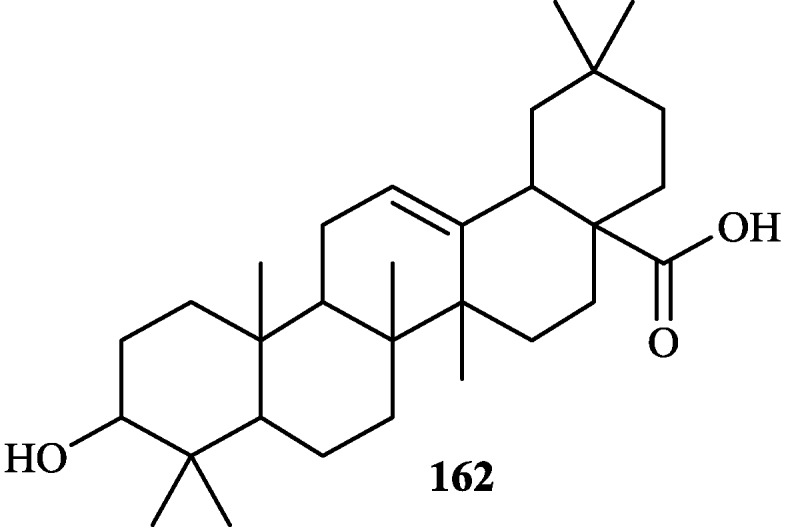
Triterpenoid from genus *Tephrosia*.

**Figure 9:**
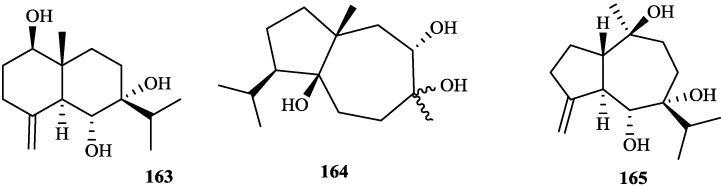
Sesquiterpenes from genus *Tephrosia*.

**Figure 10:**
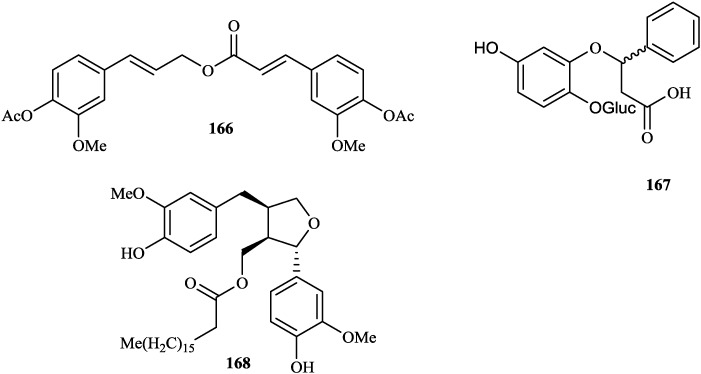
Other compounds from genus *Tephrosia*.

The chemical constituents of the genus *Tephrosia* reported since 1910 (compounds **1**–**168**) are shown in Table 1 and Figure 1, Figure 2, Figure 3, Figure 4, Figure 5, Figure 6, Figure 7, Figure 8, Figure 9, and Figure 10 below with their names, and their biological sources. As listed in the table and Figure 1, Figure 2, Figure 3, Figure 4, Figure 5, Figure 6 and Figure 7, flavonoids are the predominant constituents of this genus.

**Table 1:** Chemical constituents from the genus *Tephrosia*.

No.	Compound class and name	Source	Ref.
**Flavones**						
**1**	tephroglabrin	*T.* *purpurea*	[3]
**2**	tepurindiol	*T.* *purpurea*	[3]
**3**	glabratephrin	*T*. *apollinea*	[10]
**4**	tachrosin	*Tephrosia polystachyoides*	[17]
**5**	staohyoidin	*T*. *polystachyoides*	[18]
**6**	tephrodin	*T*. *polystachyoides*	[18]
**7**	semiglabrin	*T*. *semiglabra*, *T*. *apollinea*	[19,20]
**8**	semiglabrinol	*T*. *semiglabra*, *T*. *apollinea*	[10,19]
**9**	tephrostachin	*T*. *polystachyoides*	[21]
**10**	emoroidone	*T*. *emoroides*	[22]
**11**	tephroapollin C	*T*. *apollinea*	[23]
**12**	tephroapollin D	*T*. *apollinea*	[23]
**13**	tephroapollin E	*T*. *apollinea*	[23]
**14**	tephroapollin F	*T*. *apollinea*	[23]
**15**	tephroapollin G	*T*. *apollinea*	[23]
**16**	multijugin	*T*. *multijuga*	[24]
**17**	multijuninol	*T*. *multijuga*	[24]
**18**	pseudosemiglabrinol	*T*. *apollinea*	[25]
**19**	(−)-pseudosemiglabrin	*T*. *semiglabra*	[26]
**20**	polystachin	*T*. *polystachya*	[27]
**21**	5-methoxy-6,6-dimethylpyrano[2,3:7,6]flavone	*T*. *praecans*	[28]
**22**	candidin	*T*. *candida*	[29]
**23**	hookerianin	*T*. *hookeriana*	[30]
**24**	fulvinervin B	*T*. *fulvinervis*	[31]
**25**	fulvinervin C	*T*. *fulvinervis*	[32]
**26**	enantiomultijugin	*T*. *viciodes*	[33]
**27**	apollinine	*T*. *purpurea*	[34]
**28**	demethylapollinin 7-*O*-*β*-D-glucopyranoside	*T*. *cinerea*	[35]
**29**	tephropurpulin A	*T*. *apollinea*, *T*. *purpurea*	[36,37]
**30**	isoglabratephrin	*T*. *purpurea*	[37]
**31**	terpurinflavone	*T*. *purpurea*	[38]
**Flavonols**						
**32**	6-hydroxykaempferol 6-methyl ether 3-*O*-*α*-rhamno-pyranosyl(7→6)-*β*-galactopyranoside-7-*O*-*α*-rhamno-pyranoside	*T*. *vogelii*	[1]
**33**	6-hydroxykaempferol 6-methyl ether 3-*O*-*α*-rhamno-pyranosyl(1→2)[*α*-rhamnopyranosyl(1→6)-*β*-galacto-pyranoside	*T*. *vogelii*	[1]
**34**	6-hydroxykaempferol 6-methyl ether 3-*O*-*α*-rhamno-pyranosyl(1→2)[*α*-rhamnopyranosyl(1→ 6)]-*β*-galacto-pyranoside-7-*O*-*α*-rhamnopyranoside	*T*. *vogelii*	[1]
**35**	6-hydroxykaempferol 6-methyl ether 3-*O*-*α*-rhamnopyranosyl (1→2)[(3-*O*-*E*-feruloyl)-*α*-rhamnopyranosyl(1→6)]-*β*-galacto-pyranosides	*T*. *vogelii*	[1]
**36**	6-hydroxykaempferol 4'-methyl ether	*T*. *candida*	[39]
**37**	candidol		[40]
**38**	candirone	*T*. *candida*	[41,42]
**39**	7-ethoxy-3,3',4'-trihydroxyflavone	*T*. *procumbens*	[43]
**Flavanonols**						
**40**	(2*R*,3*R*)-3-hydroxy-5-methoxy-6'',6''-dimethylpyrano-[2'',3'':7,8]flavanone	*T*. *vogelii*	[1]
**41**	lupinifolinol	*T*. *lupinifolia*	[44]
**42**	lupinifolinol triacetate	*T*. *lupinifolia*	[44]
**Flavans**						
**43**	(2*S*)-4'-hydroxy-5-methoxy-6'',6''-dimethylpyrano[2'',3'':7,8]-flavanone	*T*. *vogelii*	[1]
**44**	(2*S*)-7-hydroxy-5-methoxy-8-prenylflavanone	*T*. *vogelii*	[1]
**45**	(2*S*)-5-methoxy-6'',6''-dimethy1-4'',5''-dihydrocyclopropa-[4'',5'']furano[2'',3'':7,8]flavanone	*T*. *vogelii*	[1]
**46**	(2*S*)-5,7-dimethoxy-8-(3-methylbut-1,3-dienyl)flavanone	*T*. *vogelii*	[1]
**47**	tephrocandidin A	*T*. *candida*	[2]
**48**	tephrocandidin B	*T*. *candida*	[2]
**49**	(+)-tephrorin A	*T*. *purpurea*	[4]
**50**	(+)-tephrorin B	*T*. *purpurea*	[4]
**51**	(2*S*)-5-hydroxy-7,4'-di-*O*-(*γ*,*γ*-dimethylallyl)flavanone	*T*. *calophylla*	[6]
**52**	6-hydroxy-*E*-3-(2,5-dimethoxybenzylidine)-2',5'-dimethoxyflavanone	*T*. *calophylla*	[6]
**53**	pumilanol	*T*. *pumila*	[13]
**54**	emoroidenone	*T*. *emoroides*	[22]
**55**	tephroapollin A	*T*. *apollinea*	[23]
**56**	tephroapollin B	*T*. *apollinea*	[23]
**57**	fulvinervin A	*T*. *fulvinervis*	[30]
**58**	lupinifolin	*T*. *lupinifolia*	[44]
**59**	5,4'-*O*,*O*-dimethyl-lupinifolin	*T*. *lupinifolia*	[44]
**60**	lupinifolin diacelate	*T*. *lupinifolia*	[44]
**61**	obovatin	*T*. *obovata*	[45]
**62**	obovatin methyl-ether	*T*. *obovata*	[45]
**63**	methylhildardtol B	*T*. *hildebrandtii*	[46]
**64**	hildgardtol B	*T*. *hildebrandtii*	[46]
**65**	hildgardtene	*T*. *hildebrandtii*	[46]
**66**	methylhildgardtol A	*T*. *hildebrandtii*	[46]
**67**	hildgardtol A	*T*. *hildebrandtii*	[46]
**68**	purpurin	*T*. *purpurea*	[47]
**69**	tephrinone	*T*. *villosa*	[48]
**70**	5,7-dimethoxy-8-prenylflavan	*T*. *madrensis*	[49]
**71**	tephrowatsin A	*T*. *watsoniana*	[50]
**72**	tephrowatsin C	*T*. *watsoniana*	[50]
**73**	tephrowatsin B	*T*. *watsoniana*	[50]
**74**	tephrowatsin D	*T*. *watsoniana*	[50]
**75**	tephrowatsin E	*T*. *watsoniana*	[50]
**76**	nitenin	*T*. *nitens*	[51]
**77**	falciformin	*T*. *falciformis*	[52]
**78**	candidone	*T*. *candida*	[53]
**79**	quercetol A	*T*. *quercetorum*	[54]
**80**	quercetol B	*T*. *quercetorum*	[54]
**81**	quercetol C	*T*. *quercetorum*	[54]
**82**	5,7-dimethoxy-8-(2,3-epoxy-3-methylbutyl)-flavanone	*T*. *hamiltonii*	[55]
**83**	tephroleocarpin A	*T*. *leiocarpa*	[56]
**84**	tephroleocarpin B	*T*. *leiocarpa*	[56]
**85**	spinoflavanone A	*T*. *spinosa*	[57]
**86**	spinoflavanone B	*T*. *spinosa*	[57]
**87**	maxima flavanone A	*T*. *maxima*	[58]
**88**	tepicanol A	*T*. *tepicana*	[59]
**89**	crassifolin	*T*. *crassifolia*	[60]
**90**	astraciceran	*T*. *strigosa*	[61]
**91**	(+)-apollineanin	*T*. *apollinea*	[62]
**92**	(2*S*)-5,4'-dihydroxy-7-*O*-[*E*-3,7-dimethyl-2,6-octadienyl]flavanone	*T*. *villosa*	[63]
**Isoflavones**			
**93**	(2*S*)-5,4'-dihydroxy-7-*O*-[*E*-3,7-dimethyl-2,6-octa-dienyl]-8-*C*-[*E*-3,7-dimethyl-2,6-octadienyl]flavanone	*T*. *villosa*	[63]
**94**	7,4'-dihydroxy-3',5'-dimethoxyisoflavone	*T*. *purpurea*	[5]
**95**	emoroidocarpan	*T*. *emoroides*	[22]
**96**	elongatin	*T*. *elongate*	[64]
**97**	pumilaisoflavone D	*T*. *pumila*	[65]
**98**	pumilaisoflavone C	*T*. *pumila*	[65]
**99**	barbigerone	*T*. *barbigera*	[66]
**100**	4'-demethyltoxicarol isoflavone	*T*. *polyphylla*	[67]
**101**	maxima isoflavone D	*T*. *maxima*	[68]
**102**	maxima isoflavone E	*T*. *maxima*	[68]
**103**	maxima isoflavone F	*T*. *maxima*	[68]
**104**	maxima isoflavone G	*T*. *maxima*	[68]
**105**	viridiflorin	*T*. *viridiflora*	[69]
**106**	maxima isoflavone J	*T*. *maxima*	[70]
**107**	pumilaisoflavone A	*T*. *pumila*	[71]
**108**	pumilaisoflavone B	*T*. *pumila*	[71]
**109**	7-*O*-geranylbiochanin A	*T*. *tinctoria*	[72]
**110**	5,7-di-*O*-prenylbiochanin A	*T*. *tinctoria*	[73]
**111**	toxicarol	*T*. *toxicaria*	[74]
**112**	villosinol	*T*. *villosa*	[75]
**113**	villosol	*T*. *villosa*	[75]
**114**	villosin	*T*. *villoss*	[76]
**115**	villol	*T*. *villoss*	[76]
**116**	villosone	*T*. *villoss*	[76]
**117**	villinol	*T*. *villoss*	[76]
**118**	dehydrodihydrorotenone	*T*. *candida*	[77]
**119**	dihydrostemonal	*T*. *pentaphylla*	[78]
**120**	9-demethyldihydrostemonal	*T*. *pentaphylla*	[78]
**121**	6-acetoxydihydrostemonal	*T*. *pentaphylla*	[78]
**122**	6a,12a-dehydro-2,3,6-trimethoxy-8-(3',3'-dimethylallyl)-9,11-dihydroxyrotenone	*T*. *villosa*	[79]
**123**	12a-dehydro-6-hydroxysumatrol	*T*. *villosa*	[80]
**124**	12a-hydroxyrotenone	*T*. *uniflora*	[81]
**125**	12a-hydroxy-*β*-toxicarol	*T*. *candida*	[82]
**126**	tephrosol	*T*. *villosa*	[83]
**127**	tephrocarpin	*T*. *bidwilli*	[84]
**128**	hildecarpin	*T*. *hildebrandtii*	[85,86]
**129**	hildecarpidin	*T*. *hildebrandtii*	[87]
**130**	2-methoxy-3,9-dihydroxy coumestone	*T*. *hamiltonii*	[88]
**131**	3,4:8,9-dimethylenedioxypterocarpan	*T*. *aequilata*	[89]
**132**	tephcalostan	*T*. *calophylla*	[90]
**133**	tephcalostan B	*T*. *calophylla*	[91]
**Chalcones**			
**134**	tephcalostan C	*T*. *calophylla*	[91]
**135**	tephcalostan D	*T*. *calophylla*	[91]
**136**	candidachalcone	*T*. *candida*	[2]
**137**	*O*-methylpongamol	*T*. *purpurea*	[3]
**138**	(+)-tephrosone	*T*. *purpurea*	[4]
**139**	(+)-tephropurpurin	*T*. *purpurea*	[5]
**140**	2',6'-dimethoxy-4',5'-(2''2''dimethyl)-pyranochalcone	*T*. *pulcherrima*	[7]
**141**	(*S*)-elatadihydrochalcone	*T*. *elata*	[14]
**142**	purpuritenin	*T*. *purpurea*	[15]
**143**	praecansone A	*T*. *praecans*	[28]
**144**	praecansone B	*T*. *praecans*	[28]
**145**	obovatachalcone	*T*. *obovata*	[45]
**146**	spinochalcone C	*T*. *spinosa*	[57]
**147**	crassichalone	*T*. *crassifolia*	[60]
**148**	oaxacacin	*T*. *woodii*	[92]
**149**	6'-demethoxypraecansone B	*T*. *purpurea*	[93]
**150**	tephrone	*T*. *candida*	[94]
**151**	spinochalcone A	*T*. *spinosa*	[95]
**152**	spinochalcone B	*T*. *spinosa*	[95]
**153**	3',5'-diisopentenyl-2',4'-dihydroxychalcone	*T*. *spinosa*	[96]
**154**	tunicatachalcone	*T*. *tunicate*	[97]
**155**	epoxyobovatachalcone	*T*. *carrollii*	[98]
**156**	2',6'-dihydroxy-3'-prenyl-4'-methoxy-*β*-hydroxychalcone	*T*. *major*	[99]
**Other Flavonoids**			
**157**	purpureamethied	*T*. *purpurea*	[15]
**158**	calophione A	*T*. *calophylla*	[91]
**159**	tephrospirolactone	*T*. *candida*	[100]
**160**	tephrospiroketone I	*T*. *candida*	[100]
**161**	tephrospiroketone II	*T*. *candida*	[100]
**Triterpenoid**			
**162**	oleanolic acid	*T*. *strigosa*	[61]
**Sesquiterpenes**			
**163**	1*β*-hydroxy-6,7*α*-dihydroxyeudesm-4(15)-ene	*T*. *candida*	[2]
**164**	linkitriol	*T*. *purpurea*	[34]
**165**	1*β*,6*α*,10*α*-guai-4(15)-ene-6,7,10-triol	*T*. *vogelii*	[101]
**Others**			
**166**	2-propenoic acid, 3-(4-(acetyloxy) -3-methoxypheny)-3(4-actyloxy)-3-methoxyphenyl)-2-propenyl ester	*T*. *purpurea*	[34]
**167**	cineroside A	*T*. *cinerea*	[35]
**168**	(+)-lariciresinol-9'-stearate	*T*. *vogelii*	[101]

### 2.1. Flavonoids

Flavonoids were the most main constituents of the genus *Tephrosia*, even of the Leguminosae family. From the year of 1971, 161 flavonoids isolated from the genus *Tephrosia* are divided into several categories depending on their skeletons (Figure 1, Figure 2, Figure 3, Figure 4, Figure 5, Figure 6 and Figure 7).

#### 2.1.1. Flavones

Thirty-one flavones (**1**–**31**), were isolated from *T*. *polystachyoides*, *T*. *semiglabra*, *T*. *multijuga*, *T*. *polystachya*, *T. praecans*, *T. apollinea*, *T. candida*, *T. purpurea*, *T. fulvinervis*, *T. viciodes*, *T. emoroids* and *T. hookeriana* [3,10,17,18,19,20,21,22,23,24,25,26,27,28,29,30,31,32,33,34,35,36,37,38].

#### 2.1.2. Flavonols

Eight flavonols (**32**–**39**), were isolated, four, *i.e.*, **32**–**34** were obtained from *T*. *vogelii* [1], one, *i.e.*, **35**–**38**, from *T*. *candida* [39,40,41,42] and **39** from *T*. *procumbens* [43].

#### 2.1.3. Flavanonols

Only three flavanonols, **40**, **41** and **42** were isolated from *T*. *vogelii* and *T*. *lupinifolia*, respectively [1,44].

#### 2.1.4. Flavans

Fifty-one flavans, **43**–**93**, were isolated from twenty-three species of the genus Tephrosia, *i.e.*, *T. obovata*, *T. villosa*, *T. madrensis*, *T. nitens*, *T. watsoniana*, *T. hildebrandtii*, *T. falciformis*, *T. hamiltonii*, *T. quercetorum*, *T. leiocarpa*, *T. spinosa*, *T. maxima*, *T. emoroides*, *T. tepicana*, *T. crassifolia*, *T. strigosa*, *T. pumila*, *T. calophylla*, *T. vogelii*, *T. apollinea*, *T. candida*, *T. purpurea* and *T. fulvinervis* [1,2,4,6,13,22,23,44,45,46,47,48,49,50,51,52,53,54,55,56,57,58,59,60,61,62,63].

#### 2.1.5. Isoflavones

Forty-two isoflavones, **94**–**135**, have been isolated and identified from this genus [5,22,64,65,66,67,68,69,70,71,72,73,74,75,76,77,78,79,80,81,82,83,84,85,86,87,88,89,90,91]. Among them, **111**–**125** were identified as rotenoids [74,75,76,77,78,79,80,81,82], **94** and **126**–**135** were identified as coumestan derivatives [22,83,84,85,86,87,88,89,90,91].

#### 2.1.6. Chalcones

Twenty-one chalcones, **136**–**156**, isolated from twelve species of genus *Tephrosia*, *i.e.*, * T*. *obovata*, *T*. *praecans*, *T*. *purpurea*, *T*. *candida*, *T*. *woodii*, *T*. *spinosa*, *T*. *crassifolia*, *T*. *tunicate*, *T*. *carrollii*, *T*. *major*, *T*. *pulcherrima* and *T*. *elata* [2,3,4,5,7,14,15,28,45,57,60,92,93,94,95,96,97,98,99].

#### 2.1.7. Other Flavonoids

**157** was isolated from *T*. *purpurea* seeds [15]. **158** was isolated from *T*. *calophylla* [91]. **159**–**161** were isolated from *T*. *candida* [100].

### 2.2. Triterpenoid

Only one triterpenoid has been isolated from this genus, that is **162** from *T*. *strigosa* [61].

### 2.3. Sesquiterpenes

Three sesquiterpenes, **163**, **164** and **165** were isolated from *T*. *candida* [2], *T*. *purpurea* [33] and *T*. *vogelii* [101], respectively.

### 2.4. Others

**166**–**168** have been isolated from *T*. *purpurea* [34], *T*. *cinerea* [35] and *T*. *vogelii* [101], respectively.

## 3. Proposed Biosynthetic Pathways and Synthesis

**Scheme 1:**
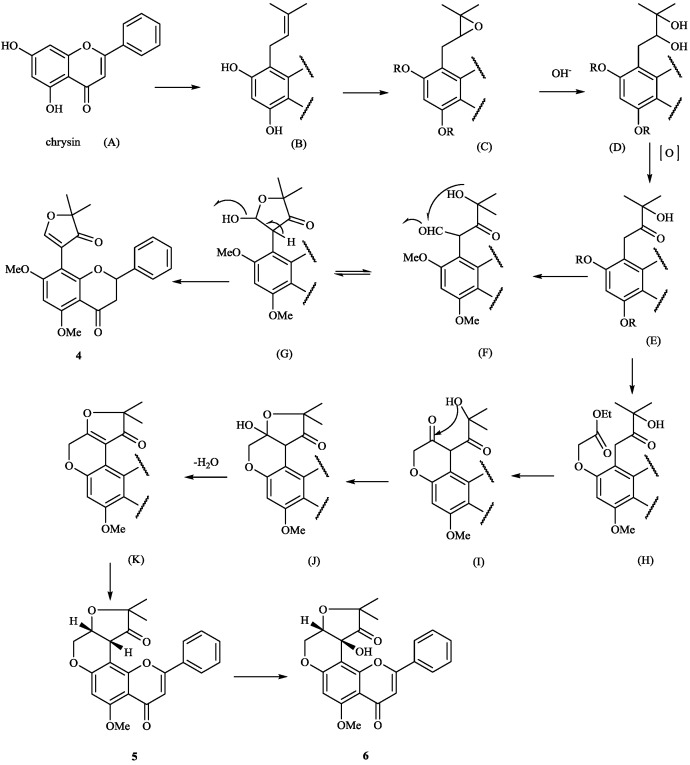
Possible biogenetic pathway of compounds **4**–**6** of *T*. *polystachyoides*.

**Scheme 2:**
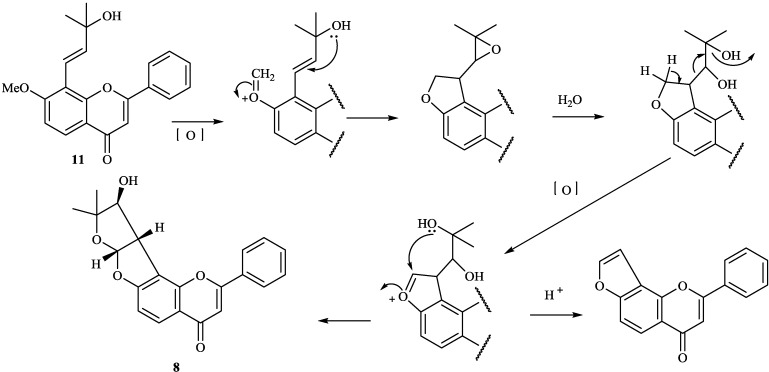
Possible biogenetic pathway of compounds **8** and **11**.

**Scheme 3:**
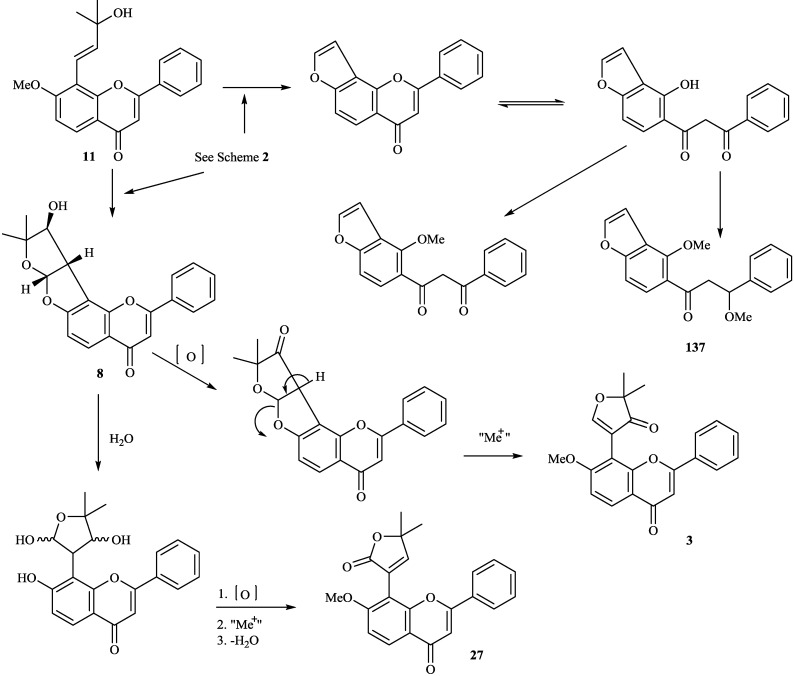
Possible biogenetic pathway of compounds **3**, **8**, **11**, **27** and **137**.

**Scheme 4:**
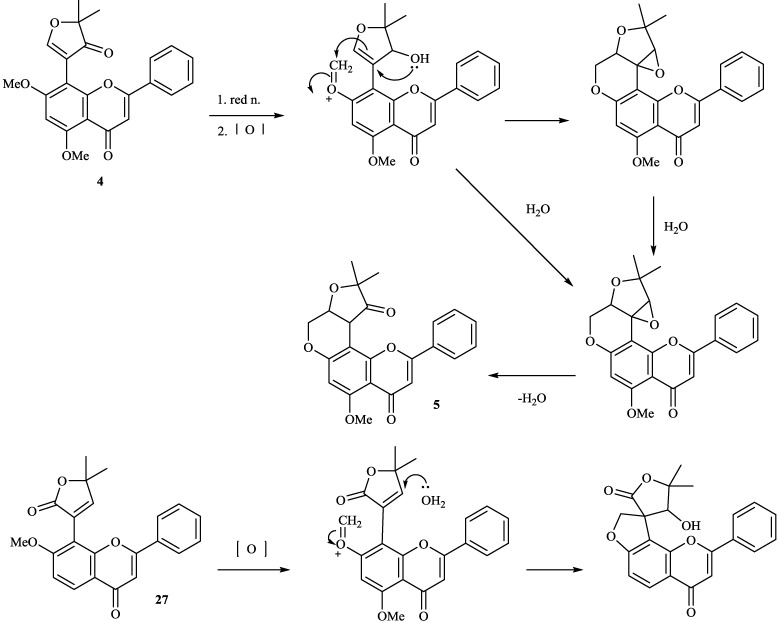
Possible biogenetic pathway of compounds **4**, **5** and **137**.

**Scheme 5:**
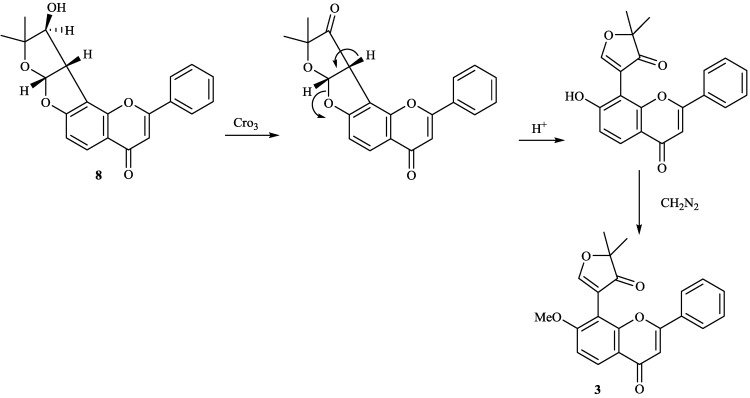
Transform of compounds **3** and **8**.

**Scheme 6:**
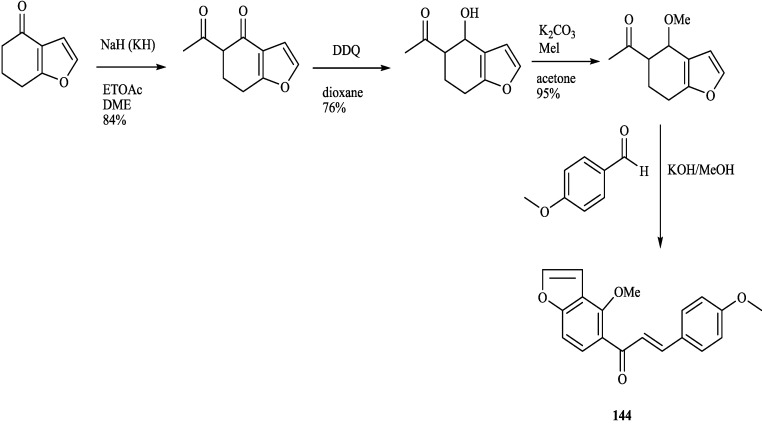
The synthesis of **144**.

8-Substituted isoflavonoids such as toxicarol isoflavone and rotenoids are well known [3]. Compounds **4**–**6** from *T*. *polystachyoides* could be explained to be evolved biogenetically from naturally occurring chrysins (A) as illustrated in the Scheme 1 [102]. It would appear that the complex substituents at C-8 arise from the ability of *Tephrosia* species to oxidise a 7-OMe group to a ‒O^+^=CH_2_ group (Scheme 2), in the same way that closely related species of Leguminosae oxidise the 2'-OMe group of isoflavonoids to yield rotenoids [103]. A pattern that explains the various C-8 substituents in *T*. *purpurea* and *T*. *apollinea* is shown in Scheme 3. In *T*. *polystachoides* this process is taken even further and the carbon of yet another 7-OMe group is incorporated into the additional rings attached to C-7 and C-8 (Scheme 4) [3]. We could confirm the structures of compounds **7** and **8** by their conversion into semiglabrinone, isoemiglabrinone and tephroglabrin (**3**) as shown in Scheme 5 [3]. Purpuritenin (**142**) was isolated from *T*. *purpurea* has been synthesed as showed in Scheme 6 [104].

## 4. Biological Activities

The chemical constituents from the genus *Tephrosia* have been shown to exhibit various bioactivities, such as estrogenic, antitumor, antimicrobial, antiprotozoal, and antifeedant activities [2,105].

### 4.1. Estrogenic Activity

Candidachalcone (**136**) isolated from *T*. *candida* exhibited estrogenic activity with IC_50_ value of 80 µM, compared with 18 µM for the natural steroid 17 *β*-estradiol [2].

### 4.2. Antitumor Activities

Calophione A (**158**) and tephcalostans B–D (**133**–**135**) from *T*. *calphylla* were evaluated for cytotoxicity against RAW (mouse macrophage cells) and HT-29 (colon cancer cells) cancer cell lines. **158** exhibited significant cytotoxicity with IC_50_ of 5.00 (RAW) and 2.90 µM (HT-29), respectively, while **133**–**135** showed moderated cytotoxicity against both RAW and HT-29 cell lines [91]. (+)-Tephrorins A (**49**) and B (**50**), and (+)-tephrosone (**138**) isolated from *T*. *purpurea* were evaluated for their potential cancer chemopreventive properties using a cell-based quinone reductase induction assay [4]. 7,4'-dihydroxy-3',5'-dimethoxyisoflavone (**94**), and (+)-tephropurpurin (**139**), were obtained as active compounds from *T*. *purpurea*, using a bioassay based on the induction of quinone reductase (QR) activity with cultured Hepa 1c1c7 mouse hepatoma cells [5].

### 4.3. Antimicrobial Activities

2',6'-Dimethoxy-4',5'-(2'',2''-dimethyl)-pyranochalcone (**140**) from *T*. *pulcherrima* showed significant antimicrobial activity when tested against a series of micro-organisms [7]. 3,4:8,9-Dimethylenedioxypterocarpan (**131**) from *T*. *aequilata* exhibited low activity against gram-positive bacteria, *Bacillus subtilis* and *Micrococcus lutea* [89]. Hildecarpin (**128**) from *T*. *hildebrandtii* had exhibited antifungal activity against *Cladosporium cucumerinum* [85,86].

### 4.4. Antiprotozoal Activities

Terpurinflavone (**31**) isolated from *T*. *purpurea* showed the highest antiplasmodial activity against the chloroquine-sensitive (D6) and chloroquine-resistant (W2) strains of *Plasmodium falciparum* with *IC*_50_ values of 3.12 ± 0.28 µM (D6) and 6.26 ± 2.66 µM (W2) [38]. The crude extract of the seedpods of *T*. *elata* showed antiplasmodial activities against D6 and W2 strains of *P*. *falciparum* with IC_50_ values of 8.4 ± 0.3 and 8.6 ± 1.0 µg/mL, respectively [14]. Obovatin (**61**) and obovatin methyl ether (**62**) from *T*. *obovata* [45] showed antiplasmodial activities against D6 and W2 strains of *P*. *falciparum* with IC_50_ values of 4.9 ± 1.7 and 6.4 ± 1.1 µg/mL, and 3.8 ± 0.3 and 4.4 ± 0.6 µg/mL, respectively [14]. (*S*)-Elatadihydrochalcone (**141**) from *T*. *elata* exhibited good antiplasmodial activity against the D6 and W2 strains of *P*. *falciparum* with IC_50_ values of 2.8 ± 0.3 (D6) and 5.5 ± 0.3 µg/mL (W2), respectively [14]. Tephcalostans C (**134**) and D (**135**) from *T*. *calphylla* were found to be weakly antiprotozoal activity *in vitro* [91]. Pumilanol (**53**) from *T*. *pumila* exhibited significant antiprotozoal activity against *T*. *b*. *rhodensiense*, *T*. *cruzi* and *L*. *donovani* with IC_50_ of 3.7, 3.35 and 17.2 µg/mL, respectively, but displayed high toxicity towards L-6 (IC_50_ of 17.12 µg/mL) rat skeletal myoblasts [13]. Tephrinone (**69**) from * T*. *villosa* [48] also exhibited high degree of activity and selectivity against both *T*. *b*. *rhodensiense*, *T*. *cruzi* and *L*. *donovani* with IC_50_ of 3.3 and 16.6 µg/mL [13].

### 4.5. Antifeedant Activities

Emoroidenone (**54**) from *T*. *emoroides* showed strong feeding deterrent activity against *Chilo partellus* larvae with a mean percentage deterrence of 66.1% at a dose of 100 µg/disc [22]. Hildecarpin (**128**) from *T*. *hildebrandtii* had exhibited insect antifeedant activity against the legume pod-borer *Maruca testulalis*, and important pest of cowpea (*Vigna*) [85,86].

### 4.6. Other Activities

(−)-Pseudosemiglabrin (**19**) from *T*. *semiglabra* displayed *in vitro* inhibitory effects on human platelet aggregation [26]. Obovatin (**61**), obovatin methyl-ether (**62**) and obovatachalcone (**145**) from *T*. *obovata* displayed moderate piscicidal activity against loach fish *Misgurnus angullicaudatus*. The TLm (median tolerance limit) values of **61**, **62** and **145** were 1.25, 1.55 and 1.35 ppm, respectively [45]. Toxicarol (**111**) was a constituent of the South American fish poison *T*. *toxicaria* [74].

## 5. Conclusions

The genus *Tephrosia*, including *ca*. 400 species, with *ca*. 52 species being investigated worldwide, was reported to possess various chemical constituents and to display diverse bioactivities, especially antiplasmodial, estrogenic, antitumor, antimicrobial, antiprotozoal, antifeedant activities. Although the number of natural compounds was isolated from this genus, there are still many *Tephrosia* species that received no little attention further, phytochemical and biological studies on this genus are needed in the future. In addition, the biosynthetic pathways and synthesis of these bioactive molecules in the genus remained largely unexplored. Thus, much more chemical, biosynthetic, synthetic and biological studies should be carried out on natural compounds in *Tephrosia* species in order to disclose their potency, selectivity, toxicity, and availability.

## References

[1] Stevenson P.C., Kite G.C., Lewis G.P., Forest F., Nyirenda S.P., Belmain S.R., Sileshi G.W., Veitch N.C. (2012). Distinct chemotypes of *Tephrosia vogelii* and implications for their use in pest control and soil enrichment. Phytochemistry.

[2] Hegazy M.E.F., Mohamed A.E.H., El-Halawany A.M., Djemgou P.C., Shahat A.A., Pare P.W. (2011). Estrogenic activity of chemical constituents from *Tephrosia candida*. J. Nat. Prod..

[3] Pelter A., Ward R.S., Rao E.V., Raju N.R. (1981). 8-Substituted flavonoids and 3'-substituted 7-oxygenated chalcones from *Tephrosia purpurea*. J. Chem. Soc. Perkin Trans. 1.

[4] Chang L.C., Chavez D., Song L.L., Farnsworth N.R., Pezzuto J.M., Kinghorn A.D. (2000). Absolute configuration of novel bioactive flavonoids from *Tephrosia purpurea*. Org. Lett..

[5] Chang L.C., Gerhauser C., Song L., Farnsworth N.R., Pezzuto J.M., Kinghorn A.D. (1997). Activity-guided isolation of constituents of *Tephrosia purpurea* with the potential to induce the phase II enzyme, quinone reductase. J. Nat. Prod..

[6] Reddy R.V.N., Khalivulla S.I., Reddy B.A.K., Reddy M.V.B., Gunasekar D., Deville A., Bodo B. (2009). Flavonoids from *Tephrosia calophylla*. Nat. Prod. Commun..

[7] Ganapaty S., Srilakshmi G.V.K., Pannakal S.T., Laatsch H. (2008). A pyranochalcone and prenylflavanones from *Tephrosia pulcherrima* (Baker) drumm. Nat. Prod. Commun..

[8] Kassem M.E.S., Sharaf M., Shabana M.H., Saleh N.A.M. (2006). Bioactive flavonoids from *Tephrosia purpurea*. Nat. Prod. Commun..

[9] Clarke G., Banerjee S.C. (1910). A glucoside from *Tephrosia purpurea*. J. Chem. Soc..

[10] Waterman P.G., Khalid S.A. (1980). The major flavonoids of the seed of *Tephrosia apollinea*. Phytochemistry.

[11] Kole R.K., Satpathi C., Chowdhury A., Ghosh M.R., Adityachaudhury N. (1992). Isolation of amorpholone, a potent rotenoid insecticide from *Tephrosia candida*. J. Agric. Food Chem..

[12] Sanchez I., Gomez-Garibay F., Taboada J., Ruiz B.H. (2000). Antiviral effect of flavonoids on the dengue virus. Phytother. Res..

[13] Ganapaty S., Pannakal S.T., Srilakshmi G.V.K., Lakshmi P., Waterman P.G., Brun R. (2008). Pumilanol, an antiprotozoal isoflavanol from *Tephrosia pumila*. Phytochem. Lett..

[14] Muiva L.M., Yenesew A., Derese S., Heydenreich M., Peter M.G., Akala H.M., Eyase F., Waters N.C., Mutai C., Keriko J.M. (2009). Antiplasmodial beta-hydroxydihydrochalcone from seedpods of *Tephrosia elata*. Phytochem. Lett..

[15] Sinha B., Natu A.A., Nanavati D.D. (1982). Prenylated flavonoids from *Tephrosia purpurea* seeds. Phytochemistry.

[16] Touqeer S., Saeed M.A., Ajaib M. (2013). A review on the phytochemistry and pharmacology of genus *Tephrosia*. Phytopharmacology.

[17] Smalberg T.M., Vleggaar R., de Waal H.L. (1971). Tachrosin: A new flavone from *Tephrosia polystachyoides* Bak F. S. Afr. J. Chem..

[18] Vleggaar R., Smalberg T.M., de Waal H.L. (1972). Two new flavones from *Tephrosia polystachyoides* Bakf 2. Tetrahedron Lett..

[19] Smalberg T.M., van den Berg A.J., Vleggaar R. (1973). Flavonoids from *Tephrosia*—VI: The structure of semiglabrin and semiglabrinol. Tetrahedron.

[20] Vleggaar R., Kruger G.J., Smalberger T.M., van den Berg A.J. (1978). Flavonoids from *Tephrosia*. XI1. Structure of glabratephrin. Tetrahedron.

[21] Vleggaar R., Smalberg T.M., de Waal H.L. (1973). Flavonoids from *Tephrosia*. V. Structure of tephrostachin. S. Afr. J. Chem..

[22] Machocho A.K., Lwande W., Jondiko J.I., Moreka L.V.C., Hassanali A. (1995). Threenew flavonoids from the root of *Tephrosia emoroides* and their antifeedant activity against the larvae of the spotted stalk Borer Chilo-Partellus Swinhoe. Pharmaceut. Biol..

[23] El-Razek M.H.A., Mohamed A.E.H.H., Ahmed A. (2007). Prenylated flavonoids, from *Tephrosia apollinea*. Heterocycles.

[24] Vleggaar R., Smalberger T.M., van den Berg A.J. (1975). Flavonoids from *Tephrosia*. IX. Structure of multijugin and multijuginol. Tetrahedron.

[25] Ahmad S. (1986). Natural occurrence of *Tephrosia* flavones. Phytochemistry.

[26] Jonathan L.T., Gbeassor M., Che C.T., Fong H.H.S., Farnsworth N.R., Lebreton G.C., Venton D.L. (1990). Pseudosemiglabrin, a platelet-aggregation inhibitor from *Tephrosia semiglabra*. J. Nat. Prod..

[27] Vleggaar R., Smalberger T.M., van Aswegen J.L. (1978). Flavonoids from *Tephrosia*. X. Structure of polystachin. S. Afr. J. Chem..

[28] Camele G., Dellemonache F., Dellemonache G., Marinibettolo G.B. (1980). Three new flavonoids from *Tephrosia praecans*. Phytochemistry.

[29] Chibber S.S., Dutt S.K. (1981). Candidin, a pyranoflavone from *Tephrosia candida* seeds. Phytochemistry.

[30] Prabhakar P., Vanangamudi A., Gandhidasan R., Raman P.V. (1996). Hookerianin: A flavone from *Tephrosia hookeriana*. Phytochemistry.

[31] Rao E.V., Venkataratnam G., Vilain C. (1985). Flavonoids from *Tephrosia fulvinervis*. Phytochemistry.

[32] Venkataratnam G., Rao E.V., Vilain C. (1986). Fulvinervin C, a flavone from *Tephrosia fulvinervis*. Phytochemistry.

[33] Gomezgaribay F., Quijano L., Hernandez C., Rios T. (1992). Flavonoids from *Tephrosia* species. IX. Enantiomultijugin, a flavone from *Tephrosia viciodes*. Phytochemistry.

[34] Khalafalah A.K., Yousef A.H., Esmail A.M., Abdelrazik M.H., Hegazy M.E., Mohamed A.E. (2010). Chemical constituents of *Tephrosia purpurea*. Pharmacogn. Res..

[35] Maldini M., Montoro P., Macchia M., Pizza C., Piacente S. (2011). Profiling of phenolics from *Tephrosia cinerea*. Planta Med..

[36] Khalafallah A.K., Suleiman S.A., Yousef A.H., El-kanzi N.A.A., Mohamed A.E.H.H. (2009). Prenylated flavonoids from *Tephrosia apollinea*. Chin. Chem. Lett..

[37] Hegazy M.E.F., Abd El-Razek M.H., Nagashima F., Asakawa Y., Pare P.W. (2009). Rare prenylated flavonoids from *Tephrosia purpurea*. Phytochemistry.

[38] Juma W.P., Akala H.M., Eyase F.L., Muiva L.M., Heydenreich M., Okalebo F.A., Gitu P.M., Peter M.G., Walsh D.S., Imbuga M. (2011). Terpurinflavone: An antiplasmodial flavone from the stem of *Tephrosia purpurea*. Phytochem. Lett..

[39] Sarin J.P.S., Singh S., Garg H.S., Khanna N.M., Dhar M.M. (1976). Flavonol glycoside with anticancer activity from *Tephrosia candida*. Phytochemistry.

[40] Dutt S.K., Chibber S.S. (1983). Candidol, a flavonol from *Tephrosia candida*. Phytochemistry.

[41] Parmar V.S., Jain R., Simonsen O., Boll P.M. (1987). Isolation of candirone—A novel pentaoxygenation pattern in a naturally-occurring 2-phenyl-4H-1-benzopyran-4-one from *Tephrosia candida*. Tetrahedron.

[42] Horie T., Kawamura Y., Kobayashi T., Yamashita K. (1994). Revised structure of a natural flavone from *Tephrosia candida*. Phytochemistry.

[43] Venkataratnam G., Rao E.V., Vilain C. (1987). Flavonoids of *Tephrosia procumbens*—Revised structure for praecansone A and conformation of praecansone B. J. Chem. Soc. Perkin Trans. 1.

[44] Smalberg T.M., Vleggaar R., Weber J.C. (1974). Flavonoids from *Tephrosia*. VII: Constitution and absolute-configuration of lupinifolin and lupinifolinol, two flavanones from *Tephrosia lupinifolia* Burch (Dc). Tetrahedron.

[45] Chen Y.L., Wang Y.S., Lin Y.L., Munakata K., Ohta K. (1978). Obovatin, obovatin methyl-ether and obovatachalcone, new piscicidal flavonoids from *Tephrosia obovata*. Agric. Biol. Chem. Tokyo.

[46] Dellemonache F., Labbiento L., Marta M., Lwande W. (1986). 4-*β*-substituted flavans from *Tephrosia hildebrandtii*. Phytochemistry.

[47] Gupta R.K., Krishnamurti M., Parthasarathi J. (1980). Purpurin, a new flavanone from *Tephrosia purpurea* seeds. Phytochemistry.

[48] Rao P.P., Srimannarayana G. (1981). Tephrinone, a new flavanone from *Tephrosia villosa*. Curr. Sci. India.

[49] Gomez F., Quijano L., Garcia G., Calderon J.S., Rios T. (1983). A prenylated flavan from *Tephrosia madrensis*. Phytochemistry.

[50] Gomez F., Quijano L., Calderon J.S., Rodriquez C., Rios T. (1985). Prenylflavans from *Tephrosia watsoniana*. Phytochemistry.

[51] Gomez F., Calderon J., Quijano L., Cruz O., Rios T. (1984). Nitenin—A new flavan from *Tephrosia nitens* Beth. Chem. Ind..

[52] Khan H.A., Chandrasekharan I., Ghanim A. (1986). Falciformin, a flavanone from pods of *Tephrosia falciformis*. Phytochemistry.

[53] Ganguly A., Bhattacharyya P., Bhattacharyya A., Adityachaudhury N. (1988). Synthesis of Candidone—A new flavanone isolated from *Tephrosia candida*. Indian J. Chem. B.

[54] Gomezgaribay F., Quijano L., Calderon J.S., Morales S., Rios T. (1988). Flavonoids from *Tephrosia* species. VI. Prenylflavanols from *Tephrosia quercetorum*. Phytochemistry.

[55] Hussaini F.A., Shoeb A. (1987). A new epoxyflavanone from *Tephrosia hamiltonii*. Planta Med..

[56] Gomezgaribay F., Quijano L., Rios T. (1991). Flavonoids from *Tephrosia* species. VII. Flavanones from *Tephrosia leiocarpa*. Phytochemistry.

[57] Rao E.V., Prasad Y.R. (1993). Prenylated flavonoids from *Tephrosia spinosa*. Phytochemistry.

[58] Rao E.V., Prasad Y.R., Murthy M.S.R. (1994). A prenylated flavanone from *Tephrosia maxima*. Phytochemistry.

[59] Gomez-Garibay F., Calderon J.S., Quijano L., Tellez O., Olivares M.D., Rios T. (1997). Flavonoids from *Tephrosia* species part 8—An unusual prenyl biflavanol from *Tephrosia tepicana*. Phytochemistry.

[60] Gomez-Garibay F., Calderon J.S., Arciniega M.D., Cespedes C.L., Tellez-Valdes O., Taboada J. (1999). Flavonoids from *Tephrosia* species part 9—An unusual isopropenyldihydrofuran biflavanol from *Tephrosia crassifolia*. Phytochemistry.

[61] Rao E.V., Sridhar P. (1999). Chemical examination of *Tephrosia strigosa*. Indian J. Chem. B.

[62] Hisham A., John S., Al-Shuaily W., Asai T., Fujimoto Y. (2006). (+)-Apollineanin: A new flavanone from *Tephrosia apollinea*. Nat. Prod. Res..

[63] Madhusudhana J., Reddy R.V.N., Reddy B.A.K., Reddy M.V.B., Gunasekar D., Deville A., Bodo B. (2010). Two new geranyl flavanones from *Tephrosia villosa*. Nat. Prod. Res..

[64] Smalberger T.M., Vleggaar R., Weber J.C. (1975). Flavonoids from *Tephrosia*. VIII: Structure of elongatin, an isoflavone from *Tephrosia elongata* E Mey. Tetrahedron.

[65] Yenesew A., Dagne E., Waterman P.G. (1989). Flavonoids from the seed pods of *Tephrosia pumila*. Phytochemistry.

[66] Vilain C. (1980). Barbigerone, a new pyranoisoflavone from seeds of *Tephrosia barbigera*. Phytochemistry.

[67] Dagne E., Mammo W., Sterner O. (1992). Flavonoids of *Tephrosia polyphylla*. Phytochemistry.

[68] Rao E.V., Murthy M.S.R., Ward R.S. (1984). Nine isoflavones from *Tephrosia maxima*. Phytochemistry.

[69] Gomez F., Calderon J.S., Quijano L., Dominguez M., Rios T. (1985). Viridiflorin, an isoflavone from *Tephrosia viridiflora*. Phytochemistry.

[70] Murthy M.S.R., Rao E.V. (1985). Maxima isoflavone J: A new O-prenylated isoflavone from *Tephrosia maxima*. J. Nat. Prod..

[71] Dagne E., Dinku B., Gray A.I., Waterman P.G. (1988). Pumilaisoflavone A and Pumilaisoflavone B from the seed pods of *Tephrosia pumila*. Phytochemistry.

[72] Reddy B.A.K., Khalivulla S.I., Gunasekar D. (2007). A new prenylated isoflavone from *Tephrosia tinctoria*. Indian J. Chem. B.

[73] Khalivulla S.I., Reddy B.A.K., Gunasekar D., Blond A., Bodo B., Murthy M.M., Rao T.P. (2008). A new di-O-prenylated isoflavone from *Tephrosia tinctoria*. J. Asian Nat. Prod. Res..

[74] Clark E.P. (1930). Toxicarol. A constituent of the South American fish poison Cracca (*Tephrosia*) toxicaria. J. Am. Chem. Soc..

[75] Sarma P.N., Srimannarayana G., Rao N.V.S. (1976). Constitution of villosol and villosinol, twonew rotenoids from *Tephrosia villosa* (Linn) pods. Indian J. Chem. B.

[76] Krupadanam G.L.D., Sarma P.N., Srimannarayana G., Rao N.V.S. (1977). New C-6 oxygenated rotenoids from *Tephrosia villosa*—Villosin, villosone, villol and villinol. Tetrahedron Lett..

[77] Roy M., Bhattacharya P.K., Pal S., Chowdhuri A., Adityachaudhury N. (1987). Dehydrodihydrorotenone and flemichapparin B in *Tephrosia candida*. Phytochemistry.

[78] Dagne E., Yenesew A., Waterman P.G. (1989). Flavonoids and isoflavonoids from *Tephrosia fulvinervis* and *Tephrosia pentaphylla*. Phytochemistry.

[79] Prashant A., Krupadanam G.L.D. (1993). A new prenylated dehydrorotenoid from *Tephrosia villosa* seeds. J. Nat. Prod..

[80] Prashant A., Krupadanam G.L.D. (1993). Dehydro-6-hydroxyrotenoid and lupenone from *Tephrosia villosa*. Phytochemistry.

[81] Abreu P.M., Luis M.H. (1996). Constituents of *Tephrosia uniflora*. Nat. Prod. Lett..

[82] Andrei C.C., Viera P.C., Fernandes J.B., daSilva M.F.D.F., Fo E.R. (1997). Dimethylchromene rotenoids from *Tephrosia candida*. Phytochemistry.

[83] Rao P.P., Srimannarayana G. (1980). Tephrosol, a new coumestone from the roots of *Tephrosia villosa*. Phytochemistry.

[84] Ingham J.L., Markham K.R. (1982). Tephrocarpin, a pterocarpan phytoalexin from *Tephrosia bidwilli* and a structure proposal for acanthocarpan. Phytochemistry.

[85] Lwande W., Bentley M.D., Hassanali A. (1986). The structure of hildecarpin, an insect antifeedant 6a-hydroxypterocarpan from the roots of *Tephrosia hildebrandtii* Vatke. Int. J. Trop. Insect Sci..

[86] Lwande W., Hassanali A., Njoroge P.W., Bentley M.D., Delle Monache F., Jondiko J.I. (1985). A new 6a-hydroxypterocarpan with insect antifeedant and antifungal properties from the roots of *Tephrosia hildebrandtii* Vatke. Int. J. Trop. Insect Sci..

[87] Lwande W., Bentley M.D., Macfoy C., Lugemwa F.N., Hassanali A., Nyandat E. (1987). A new pterocarpan from the roots of *Tephrosia hildebrandtii*. Phytochemistry.

[88] Rajani P., Sarma P.N. (1988). A coumestone from the roots of *Tephrosia hamiltonii*. Phytochemistry.

[89] Tarus P.K., Machocho A.K., Lang’at-Thoruwa C.C., Chhabra S.C. (2002). Flavonoids from *Tephrosia aequilata*. Phytochemistry.

[90] Kishore P.H., Reddy M.V.B., Gunasekar D., Murthy M.M., Caux C., Bodo B. (2003). A new coumestan from *Tephrosia calophylla*. Chem. Pharm. Bull. (Tokyo).

[91] Ganapaty S., Srilakshmi G.V.K., Pannakal S.T., Rahman H., Laatsch H., Brun R. (2009). Cytotoxic benzil and coumestan derivatives from *Tephrosia calophylla*. Phytochemistry.

[92] Dominguez X.A., Tellez O., Ramirez G. (1983). Mixtecacin, a prenylated flavanone and oaxacacin its chalcone from the roots of *Tephrosia woodii*. Phytochemistry.

[93] Rao E.V., Raju N.R. (1984). Two flavonoids from *Tephrosia purpurea*. Phytochemistry.

[94] Chibber S.S., Dutt S.K. (1982). Tephrone, a new chalcone from *Tephrosia candida* seeds. Curr. Sci. India.

[95] Rao E.V., Prasad Y.R. (1992). Two chalcones from *Tephrosia spinosa*. Phytochemistry.

[96] Sharma V.M., Rao P.S. (1992). A prenylated chalcone from the roots of *Tephrosia spinosa*. Phytochemistry.

[97] Andrei C.C., Ferreira D.T., Faccione M., de Moraes L.A.B., de Carvalho M.G., Braz R. (2000). C-prenylflavonoids from roots of *Tephrosia tunicata*. Phytochemistry.

[98] Gomez-Garibay F., Arciniega M.D.O., Cespedes C.L., Taboada J., Calderon J.S. (2001). Chromene chalcones from *Tephrosia carrollii* and the revised structure of oaxacacin. Z. Naturforsch. C.

[99] Gomez-Garibay F., Tellez-Valdez O., Moreno-Torres G., Calderon J.S. (2002). Flavonoids from *Tephrosia major*. A new prenyl-*β*-hydroxychalcone. Z. Naturforsch. C.

[100] Andrei C.C., Vieira P.C., Fernandes J.B., da Silva M.F., Rodrigues Fo E. (2002). New spirorotenoids from *Tephrosia candida*. Z. Naturforsch. C.

[101] Wei H.H., Xu H.H., Xie H.H., Xu L.X., Wei X.Y. (2009). Sesquiterpenes and lignans from *Tephrosia vogelii*. Helv. Chim. Acta.

[102] Jain A.C., Gupta R.C. (1978). Possible biogenesis of novel type of flavones from *Tephrosia polystachyoides*. Curr. Sci. India.

[103] Crombie L., Dewick P.M., Whiting D.A. (1973). Biosynthesis of rotenoids—Chalcone, isoflavone, and rotenoid stages in formation of amorphigenin by *Amorpha fruticosa* seedlings. J. Chem. Soc. Perkin Trans. 1.

[104] Lee Y.R., Morehead A.T. (1995). A new route for the synthesis of furanoflavone and furanochalcone natural products. Tetrahedron.

[105] Belmain S.R., Amoah B.A., Nyirend S.P., Kamanula J.F., Stevenson P.C. (2012). Highly variable insect control efficacy of *Tephrosia vogelii* Chemotypes. J. Agric. Food Chem..

